# Aesthetic Reconstruction of Secondary Wounds after Extended Resection of Dermatofibrosarcoma Protuberans: A Retrospective Study in One Center

**DOI:** 10.1111/jocd.70217

**Published:** 2025-04-30

**Authors:** Bing‐Jie Zhou, Hai‐Yan Zhong, Yong Chen, Qian Wang, Min Wang, Ming‐Li Zou, Si‐Ming Yuan

**Affiliations:** ^1^ Southeast University School of Medicine Nanjing Jiangsu China; ^2^ Department of Plastic Surgery, Nanjing Jinling Hospital, Jinling Clinical Medical College Nanjing University of Chinese Medicine Nanjing Jiangsu China

**Keywords:** dermatofibrosarcoma protuberans, wide excision, wound repair

## Abstract

**Background:**

Dermatofibrosarcoma protuberans (DFSP) is a rare soft tissue malignancy with aggressive growth.

**Objective:**

To summarize the surgical treatment and results of DFSP based on a case series and literature review.

**Methods:**

Seventeen DFSP cases were analyzed retrospectively at our hospital from June 2016 to December 2022, including 14 males and 3 females. All these cases were treated by wide local excision with negative margins. Postoperative follow‐up was performed to observe recurrence, survival, and the effect of wound repair.

**Results:**

Routine postoperative pathology confirmed the morphological subtypes of these cases, including 12 cases of classical DFSP, 3 cases of DFSP‐fibrosarcoma, 1 case of myxoid, and 1 case of Bednar. The median defect size was 72 cm^2^ (interquartile range 20–750 cm^2^). All patients required split skin (47.1%) or local flap (52.9%) to reconstruct the soft tissue defect caused by tumor resection. All split‐skin grafts were viable, and one flap case had marginal necrosis, which healed by dressing change. After 6–32 months of follow‐up, local recurrence occurred in 1 case, and extensive intra‐abdominal metastasis and death occurred in another case.

**Conclusion:**

Wide local excision with negative margins can achieve a favorable prognosis in classical DFSP. The use of flaps for reconstruction results in better appearance and function.

## 1 Introduction

Dermatofibrosarcoma protuberans (DFSP) is a rare low‐grade malignancy that originates from the dermis or subcutis, with an incidence rate of approximately 4.5 per million [1]. Although it can happen at any age, middle‐aged individuals are more predisposed to the disease [2]. In its early stage, DFSP is characterized by slow‐growing dermal plaques or painless nodules. Misdiagnosis rates for DFSP are high due to its easy confusion with benign diseases such as “hypertrophic scar”, “keloid”, “benign mass”, etc. [2]. DFSP is distinguished by aggressive growth [3], a high rate of local recurrence, and a low potential for distant metastases. According to the characteristics of pathologic and immunohistochemical results, DFSP can be classified into 10 histological subtypes, including classic DFSP and fibrosarcomatous DFSP (DFSP‐FS), among which DFSP‐FS has higher metastatic potential [4].

The mainstay treatment for DFSP is surgical resection [5], including wide local excision (WLE) and Mohs micrographic surgery (MMS). Because of the tumor's aggressive expansion, MMS shows lower recurrence rates compared to WLE [6]. However, several studies find that MMS has no obvious advantage over WLE in terms of postoperative recurrence rates [7]. The majority of existing studies comparing the advantages and disadvantages of the two are almost retrospective small case series, with no prospective randomized controlled studies. Thus, there is no convincing evidence‐based proof of whether MMS is superior to WLE. Given that MMS is more complicated and consumes more operation time, as well as the difficulty in distinguishing which one is better than another one, WLE is more widely used at the present stage. However, WLE has to face a challenge—how to repair the large soft tissue defects after surgical resection? In this study, we retrospectively analyzed 17 cases of DFSP who were treated with WLE and reconstructive surgery from June 2016 to December 2022 in our hospital and summarized our experiences to provide reference for surgical treatment and reconstruction.

## 2 Methods

The study was a retrospective study, as the small number of cases did not justify a prospective study. The study was approved by the institutional review board of the Jinling Hospital and was conducted in accordance with the Declaration of Helsinki. Written informed consent was obtained from all patients for surgery and publication. Patients who were surgically treated in our hospital from June 2016 to December 2022 were included in the study. Patients were excluded if they were unable to undergo surgery or did not respond to surgical treatment. Data retrieved included sex, age, course of disease, anatomical location of the lesion, primary or recurrent occurrence, tumor size, pathological type, width of surgical margins, type of wound closure, postoperative complications, and use of adjuvant radiation therapy. Postoperative recurrence and/or metastasis, as well as the time of cancer‐specific deaths, were followed up via telephone, email, or outpatient visit.

**FIGURE 1:**
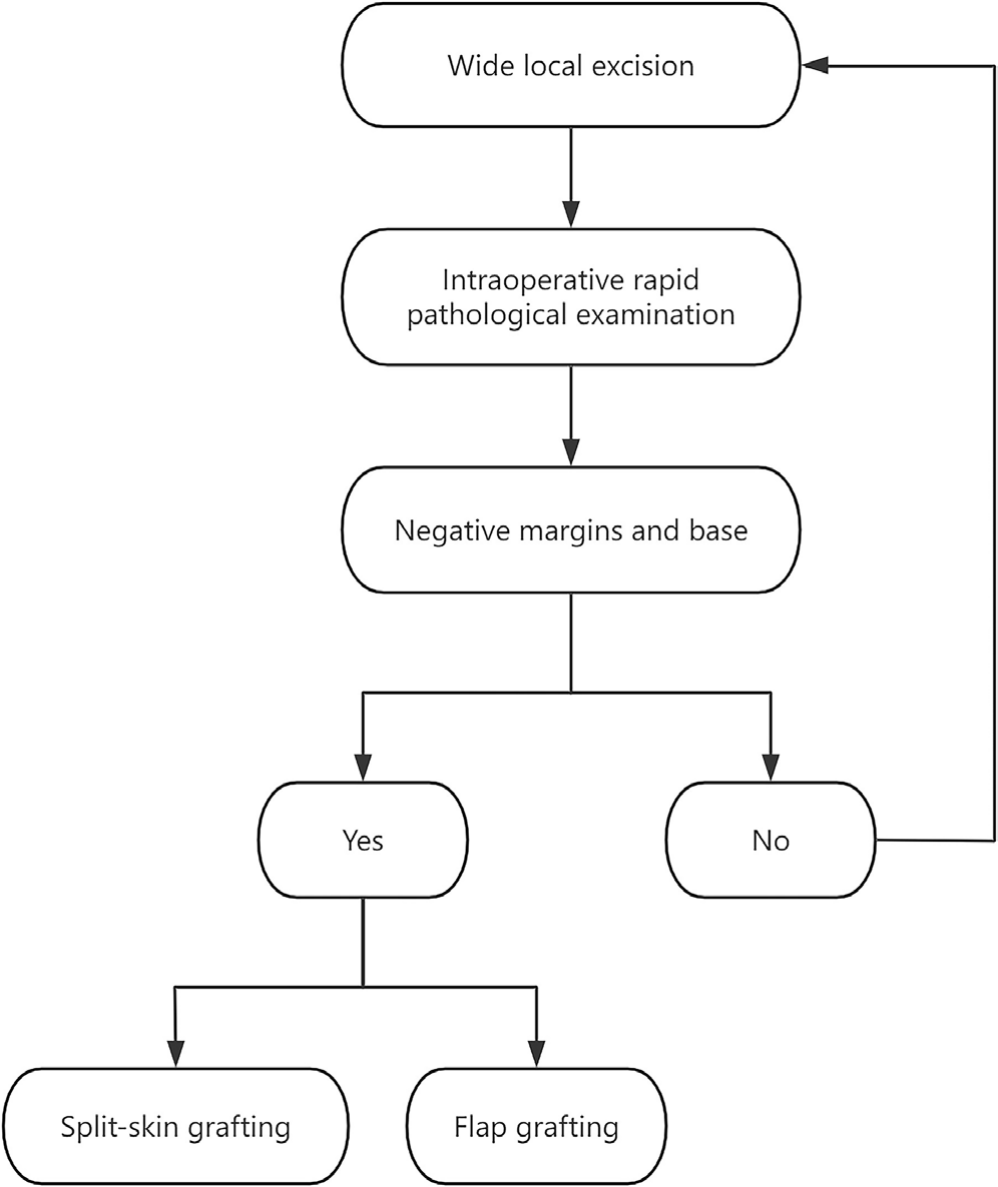
The flow chart of the surgical procedures.

Figure 1 is the flow chart of the surgical procedures. All cases in this study were treated by WLE with surgical margins of 2–3 cm. The resection region was determined by the tumor size, location, and imaging data. The tumor tissue and surrounding tissues were fully removed within the designated area. After intraoperative rapid pathological examination confirmed that the margins and base were negative, the reconstruction strategy was decided on the basis of the size and depth of the soft tissue defects, as well as the blood circulation of the bed. Split‐skin grafting is commonly used when the wound has sufficient blood supply but no critical tissue exposure such as bone or tendon, and is less aesthetically relevant. A local flap is used when important tissues such as bone, tendon, or muscle stumps are exposed or the wound is situated in aesthetically important areas. The flaps used in this study were all pedicled axial flaps, which were flexibly tailored to match the blood supply conditions surrounding the wound.

## 3 Results

### 3.1 Demographic Data

Data of all the cases were summarized in Table 1. There were 17 cases in total, 14 males (82.4%) and 3 females (17.6%), one of which had prior trauma at the site of the disease. The median age of the group was 46 years (range 21–72 years). The median course of the group was 7 years (range 1–40 years). Nine cases (52.9%) were in the trunk, 6 cases (35.3%) were in the limbs, and 2 cases (11.8%) were in the head, face, or neck. The median maximum diameter of the lesions was 5 cm (range 2–30 cm). Four cases (23.5%) were diagnosed as primary tumors; 5 cases (29.4%) had been treated with local resection and diagnosed as DFSP by pathology in another hospital, and then came to our hospital to seek extended resection within 3 months; 8 cases (47.1%) were recurrent DFSP.

**TABLE 1:** Demographic data of patients with dermatofibrosarcoma protuberans in the study.

Case#	Sex	Age (years)	Site	Size (cm)	Primary/Recurrent	Pathological types	Reconstruction	Outcome upon follow‐up
1	Male	67	Abdominal wall	30 × 25	Recurrent	DFSP‐FS	Split‐skin grafting	24 months, death
2	Male	60	Right shoulder	8 × 8	Recurrent	Classicial DFSP	Split‐skin grafting	No recurrence during follow‐up
3	Male	37	Abdominal wall	2 × 2	Recurrent	Classicial DFSP	Thigh broad fascia grafting; Flap	No recurrence during follow‐up
4	Male	62	Left shoulder	6 × 4	Expanding excision	Classicial DFSP	Split‐skin grafting	No recurrence during follow‐up
5	Male	43	Left temporal scalp	3.5 × 4	Recurrent	Classicial DFSP	Split‐skin grafting	No recurrence during follow‐up
6	Male	25	Left ribcage	4 × 4	Recurrent	Classicial DFSP	Flap	No recurrence during follow‐up
7	Male	34	Left armpit	4 × 5	Expanding excision	Classicial DFSP	Flap	No recurrence during follow‐up
8	Male	59	Right back	3 × 4	Recurrent	Fibrosarcoma	Flap	8 months, recurrence
9	Female	46	Chest	3 × 4	Recurrent	Classicial DFSP	Split‐skin grafting	No recurrence during follow‐up
10	Male	21	Frontoparietal scalp	3 × 3	Recurrent	Classicial DFSP	Flap	No recurrence during follow‐up
11	Male	49	Abdominal wall	4 × 6	Expanding excision	Classicial DFSP	Flap	No recurrence during follow‐up
12	Male	38	Chest wall	5 × 7	Primary	Classicial DFSP	Split‐skin grafting	No recurrence during follow‐up
13	Male	72	Right hallux	5 × 7	Primary	Classicial DFSP	Flap	No recurrence during follow‐up
14	Male	35	Left hip	5 × 5	Recurrent	Classicial DFSP	Split‐skin grafting	No recurrence during follow‐up
15	Male	53	Right shoulder	4 × 4	Expanding excision	Myxoid DPSP	Flap	No recurrence during follow‐up
16	Female	27	Right shoulder	2 × 2	Primary	Bednar	Split‐skin grafting	No recurrence during follow‐up
17	Female	63	Breast	6 × 4	Primary	DFSP‐FS	Flap	No recurrence during follow‐up

### 3.2 Analysis of Surgical Methods

All 17 cases in our study used WLE, and the size of soft tissue defects ranged from 4 × 5 to 25 × 30 cm. Eight cases underwent split‐skin grafting, whereas nine were treated with local flaps, and the donor site was closed primarily or repaired by split‐skin grafting. In one case, the entire abdominal wall was damaged as a result of a wide resection of the left rectus abdominis muscle tumor under the raphe. The peritoneum defect was repaired by the broad fascia of the left thigh, as well as the remaining defect was repaired by a rectus abdominis muscle flap which took the right deep superior epigastric artery as the pedicle. Among the other flap cases, one was a left paraumbilical flap, one was a left deep inferior epigastric artery flap, one was a circumflex scapular artery flap, one was a latissimus dorsi myocutaneous flap, one was a superficial temporal vessel flap, one was a dorsal pedis flap, one was an arteriae circumflexa brachialis lateralis flap, and one was the third intercostal internal mammary artery perforator flap. All details of these flaps used in our study can be found in Table 2. All split‐skin grafts were viable. One flap developed marginal necrosis, which healed with conservative wound care, including gentle debridement and regular dressing changes.

**TABLE 2:** Detailed information of each flap used in the study.

Case#	Flap name	Flap type	Soft tissue defect site	Flap anatomical site	Vascular supply
1	Deep superior epigastric artery flap	Axial flap	Upper abdominal wall	Upper right abdominal wall	Deep superior epigastric artery
2	Left paraumbilical flap	Axial flap	Left ribcage	Left abdominal wall	Umbilical perforator
3	Circumflex scapular artery flap	Axial flap	Left armpit	Lateral posterior chest wall	Circumflex scapular artery
4	Latissimus dorsi myocutaneous flap	Axial flap	Upper right back	Lower upper latissimus dorsi	Thoracodorsal artery
5	Superficial temporal vessel flap	Axial flap	Parietal region	Right temporal region	Superficial temporal vessel
6	Deep inferior epigastric artery flap	Axial flap	Lower left abdominal wall	Upper right abdominal wall	Deep inferior epigastric artery
7	Dorsalis pedis flap	Axial flap	Right hallux	Right foot dorsum	Dorsalis pedis artery
8	Arteriae circumflexa brachialis lateralis flap	Axial flap	Right shoulder	Right deltoid	Lateral circumflex brachial artery
9	The third intercostal internal mammary artery perforator flap	Axial flap	Breast	Right upper chest wall	The third intercostal perforator of internal mammary artery

### 3.3 Antitumor Therapy and Follow‐Up Results

The median time of follow‐up was 12 months (range 6–32 months). One case of dorsal DFSP was treated with radiotherapy after initial resection in another hospital, but the tumor recurred. The patient continued to be treated with radiotherapy after WLE and flap transfer in our hospital. In the early stage, no recurrence happened. However, the tumor recurred again at 8 months after the operation without distant metastasis. The patient did not continue to be treated with surgery and was still surviving with the tumor until the end of the follow‐up period. The remaining patients did not receive any antitumor therapy. One case of giant abdominal DFSP developed extensive intraperitoneal metastasis during the follow‐up period and died 24 months after surgery. The postoperative recurrence rate in our cases was 11.8% and the mortality rate was 5.9%. According to pathological subtypes, the postoperative recurrence rate was 66.7% in the DFSP‐FS group and 0% in the remaining subtypes. The postoperative recurrence rate of DFSP‐FS was much higher than the other subtypes. The color and texture of all the flaps in the study were similar to the surrounding normal skin, and the morphology, which includes tissue integration and symmetry, was good. Scars were visible at the site of split‐skin grafting and the appearance was poor.

**FIGURE 2:**
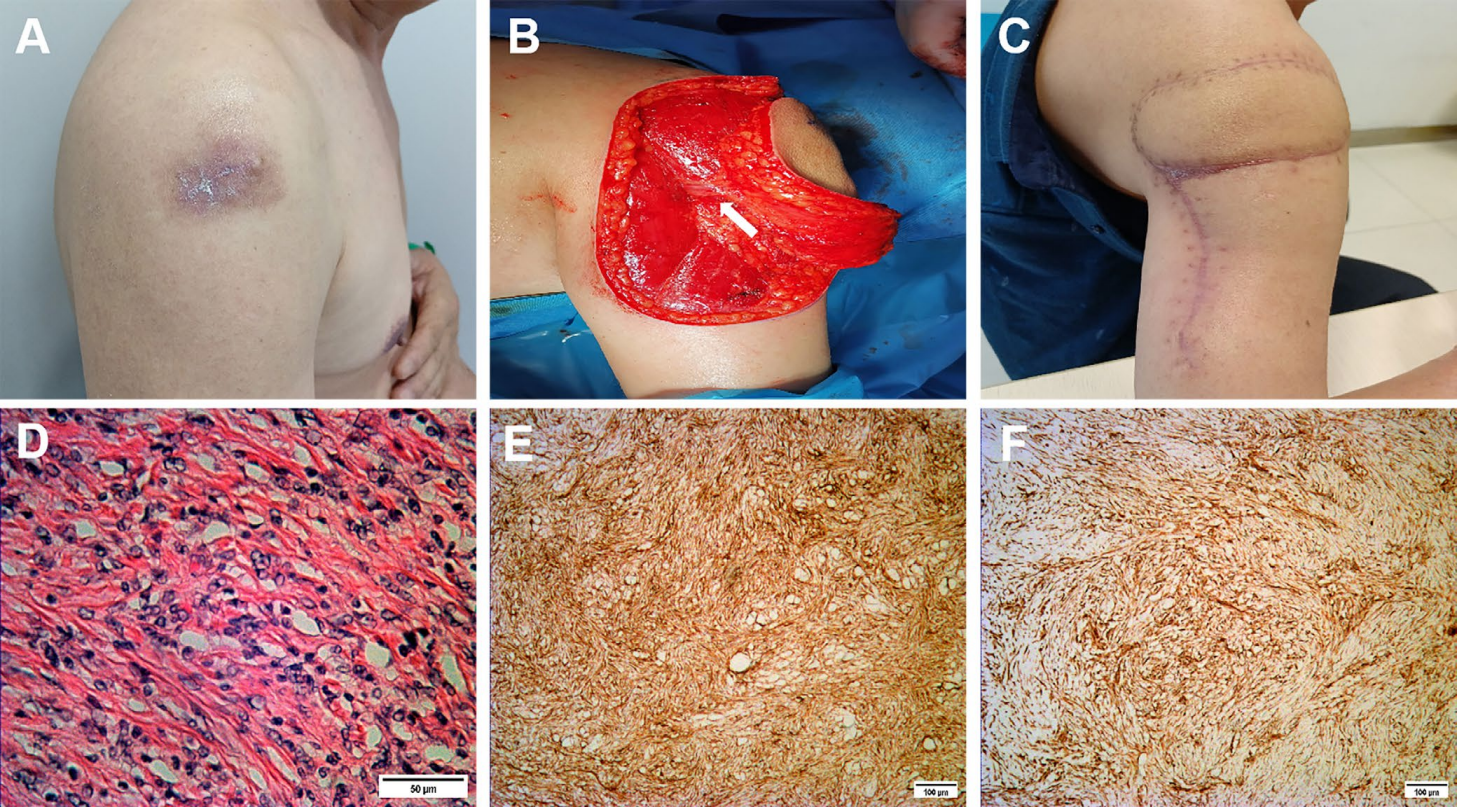
(A) DFSP removed locally 2 weeks ago in the right shoulder of a 53‐year‐old man. (B) Vascular pedicle of the arteriae circumflexa brachialis lateralis flap (white arrow). (C) 2 months after the surgery. (D) Spindle cells and interspersed thin‐walled vessels within myxoid or myxocollagenous stroma on histology (scale bar, 50 μm). (E, F) Immunohistochemistry, the tumor showed strong and diffuse expression of CD34 and Vimentin (scale bar, 100 μm).

**FIGURE 3:**
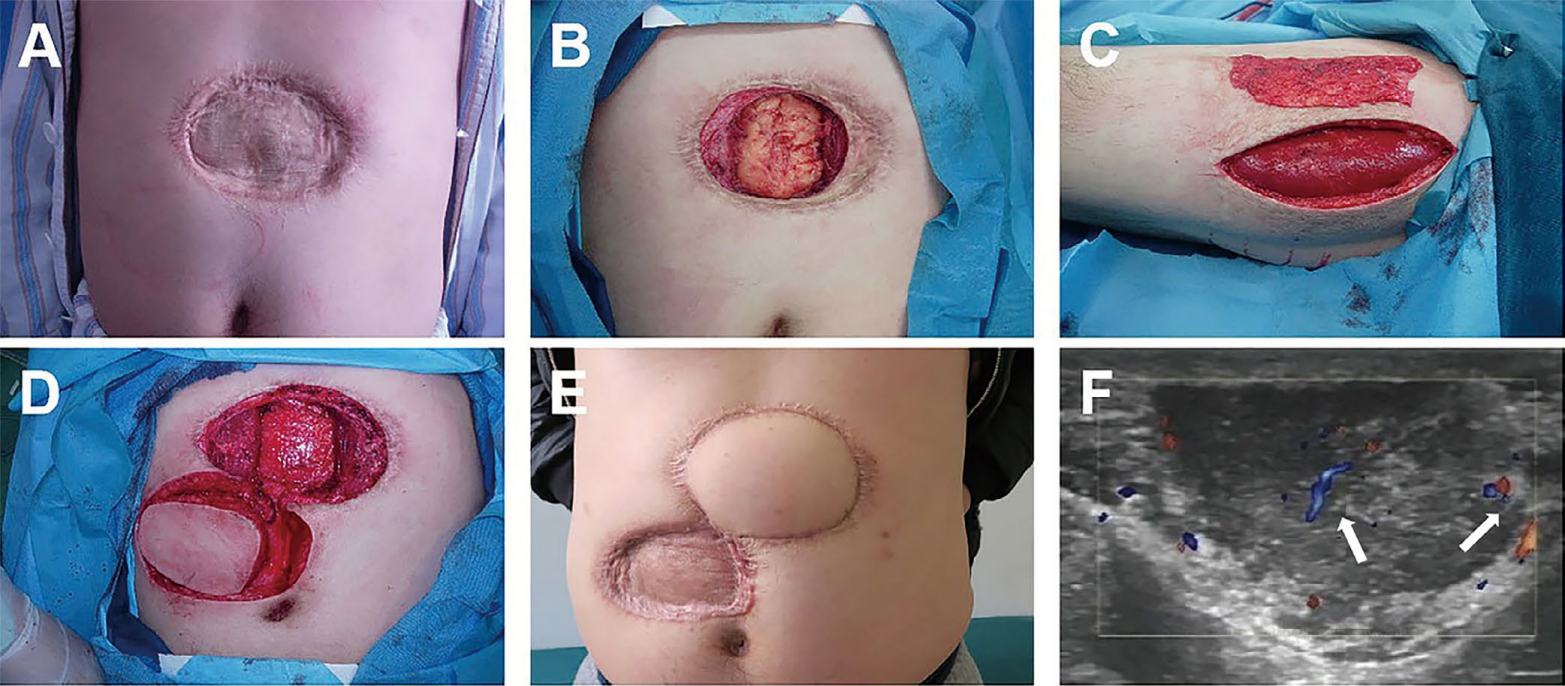
(A) A 37‐year‐old man with recurrent DFSP in the abdominal wall. (B) The peritoneum defect after removing the tumor. (C) Harvesting the broad fascia of the left thigh. (D) Harvesting the deep superior epigastric artery flap. (E) 6 months after the surgery. (F) Color Doppler ultrasonogram revealing rod‐shaped blood flow signals (white arrow).

**FIGURE 4:**
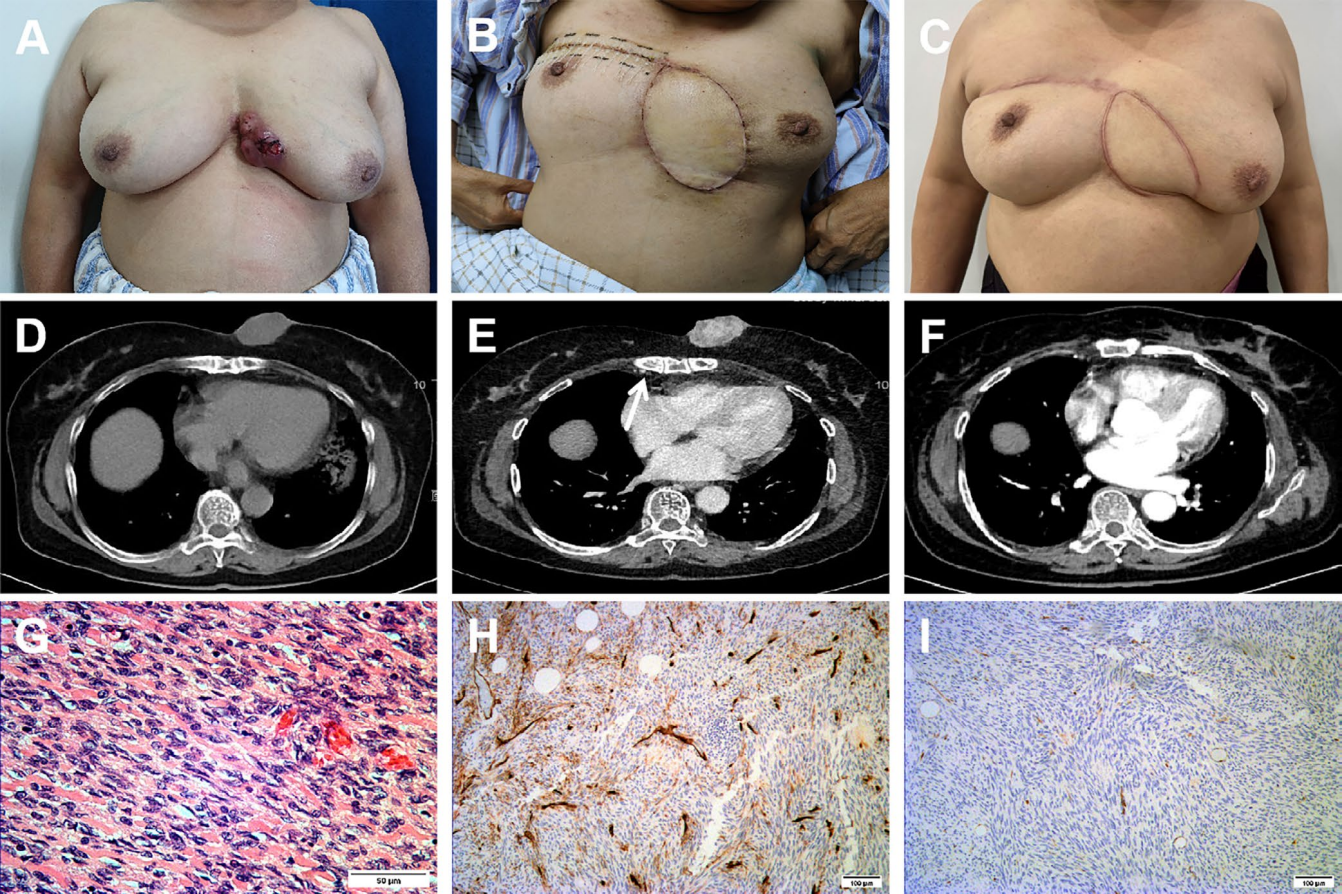
(A) Primary DFSP in the chest wall of a 63‐year‐old woman. (B) 12 days after the surgery. (C) 3 months after the surgery. (D) The tumor was iso‐dense to muscle on unenhanced CT. (E) Marked inhomogeneous enhancement and advantageous perforator (white arrow) on enhanced CT. (F) No recurrence on enhanced CT reexamination 3 months later. (G) Histology demonstrated a tumor with a fasciculated architecture (scale bar, 50 μm). (H, I) Immunohistochemistry, the tumor was weakly positive for CD34 and negative for S‐100 (scale bar, 100 μm).

Figures 2, 3, 4 show the typical cases repaired by local flaps.

## 4 Discussion

DFSP is common in adults aged 25–45 [2]; nevertheless, the actual age of onset will be younger due to the protracted course of the disease and low specificity at the early stage [8]. DFSP can affect any part of the body, preferably in the trunk (40%–50%), especially the anterior thorax and abdominal wall, followed by the limbs (30%–40%), the head and neck (10%–15%), and the perineum (1%) [1]. The clinical manifestations are mainly painless skin lumps or nodules that are hard, irregular in shape, and limited in mobility. As a result, it is easily confused with keloid. DFSP is characterized by infiltrative growth [3], the local recurrence rate is relatively high, while distant metastasis is rare.

Currently, histologic examination, often with the use of appropriate immunostains, is necessary for diagnosis. DFSP can be classified into 10 histological subtypes, including classical DFSP, DFSP‐fibrosarcomatous, Bednar, myxoid, giant cell fibroblastoma, and atrophic, etc. [2]. DFSP‐FS has the highest risk of distant metastasis [4]. Classical DFSP is the most common subtype, accounting for more than 80% of all cases [2]. Microscopically, the tumor cells are in the shape of a single spindle, which can be arranged in the form of a mat, a whirlpool, or a wheel, and if infiltrating into surrounding fat tissue, it will present a honeycomb‐like structure. Immunohistochemically, spindle cells typically show strong and diffuse cytoplasmic expression of CD34 in classical DFSP [9], whereas in fibrosarcoma DFSP, CD34 often expresses weakly positive or negative [10]. More than 95% of DFSPs are characterized by either supernumerary ring chromosomes derived from chromosomes 17 and 22 or chromosomal translocation t(17;22) (q22;q13), resulting in the fusion of collagen type I‐alpha1 (COL1A1 at 17q22) and platelet‐derived growth factor beta (PDGFB at 22q13) genes [10]. A recent study found that in addition to the COL1A1‐PDGFβ fusion gene, a novel fusion gene of SLC2A5‐BTBD7 is also present in DFSP, which may serve as a new therapeutic target [11].

In addition to histologic diagnosis, imaging also plays an important role in the differential diagnosis and preoperative evaluation of the tumor. On ultrasound, DFSP usually indicates posterior hyperechoic areas with rich‐vascular lesions [12]. On CT images, the tumors might appear as a bulging development toward the skin and hanging out of the skin, which is known as the “hanging sign” and is a very specific imaging presentation of the disease [13]. Additionally, chest CT can be utilized to judge whether lung metastasis has occurred. Magnetic resonance imaging (MRI) is the most suitable imaging modality for diagnosing soft tissue tumors because it can show the tumor size, infiltration depth, and connection with surrounding normal tissues more precisely. DFSP often shows “skin tail sign” and “fascial tail sign” on MRI images, which refers to the thickening of the skin or fascia adjacent to the tumor [14].

At present, the first choice of treatment for DFSP is surgical resection, with two primary methods—WLE and MMS. Although MMS can maximize the preservation of normal tissues, there is no scientific, reliable, and precise evidence to prove that its postoperative recurrence rate is lower than that of WLE [7]. Moreover, MMS needs a long time and high cost, which entails high technical requirements of doctors and hardware of medical institutions. In comparison, WLE is simpler, so it is more widely applied in most medical institutions. However, due to the tumor's infiltrative growth, WLE has to face the challenge of choosing the appropriate peripheral margins to realize a balance between reducing the postoperative recurrence rate and preserving as much normal tissue as possible. Although consensus guidelines for the treatment of DFSP are available, significant variations exist between healthcare systems. The US National Comprehensive Cancer Network guidelines recommend WLE with a peripheral margin of 2–4 cm or MMS [15]. European guidelines recommend MMS and slow MMS over WLE, with a peripheral safety excision margin of 1–1.3 cm. If WLE is used, a peripheral safety margin of 3 cm is recommended [10]. Besides, Danish guidelines recommend a peripheral safety margin of 2–3 cm [16]. In this study, a peripheral margin of 2–3 cm was used, and 2 cases (11.8%) occurred recurrence after the operation. Joaquim Marcoval [17] and Hiba Saifuddin [18] reported postoperative recurrence rates of 11.49% and 11.11% respectively, which are similar to our result. However, it is worth noting that both of the two cases were patients with recurrent DFSP when they came to our hospital, and the pathologic classification was DFSP‐FS. The postoperative recurrence rate of DFSP‐FS was much higher than that of the other subtypes. Additionally, the largest diameter of the tumor can reach 30 cm in the two cases. Several studies can prove some viewpoints: the prognosis for patients with recurrent DFSP is worse than for patients with primary DFSP [19], fibrosarcoma DFSP is more aggressive than other subtypes [17, 20], and larger tumors have a relatively worse prognosis [20]. Taken together, adopting WLE with a peripheral margin of at least 2 cm and pathologically negative margins based on the tumor appearance and the extent of tumor infiltration on imaging can achieve a more satisfactory prognosis.

In this study, 17 patients had soft tissue defects that measured a minimum of 4 × 5 cm and a maximum of 25 × 30 cm after tumor resection. All of the wounds can notbe closed directly. Split‐skin or flap was used according to the size and depth of the soft tissue defects. One case with a peritoneal defect was special, which was repaired first by the broad fascia of the thigh. The repair of the wounds after extended resection is a great challenge, which emphasizes the unique advantages of plastic surgeons in the treatment of DFSP. Although split‐skin grafting is easy to perform, flap can repair more complex wounds and achieve better cosmetic results. Taking together the preoperative ultrasound, MRI, and CT‐enhanced angiography (CTA) to define the depth and width of the tumor infiltration and guide the design of a reasonable resection range and appropriate repair method, especially the targeted design of the flap, is very important to improve the success rate of the surgery and achieve better prognosis.

Postoperative complications should be noted. In the study, marginal necrosis occurred in one case but healed without surgical intervention. However, some other studies [18, 21] reported more complications such as delayed wound healing, infection, painful scar that required revision, hematoma, seroma, hernia, and unplanned admission to the hospital for pain control.

Targeted therapy and radiation therapy are the two primary adjuvant therapies being used as postoperative consolidation therapy for DFSP. A study [22] found that the use of imatinib mesylate (IM) as a neoadjuvant therapy led to tumor volume shrinkage in 60% of patients, and patients with the genetic alteration of a t(17;22) (q22;q13) translocation were sensitive to the IM therapy. If IM therapy proves to be ineffective or resistant, the second‐line drugs sunitinib or pazopanib can be tried [23]. As DFSP is a radiation‐responsive tumor, the rate of postoperative recurrence can be decreased by combining radiation therapy with other treatments [24]. Currently, there are no unified standards for radiation therapy in DFSP; some guidelines recommend taking 5 times of radiation therapy per week, 2 Gy each time, with a total dose of 60–70 Gy [10]. In our study, a patient with recurrent DFSP received radiation therapy after the first resection but was ineffective. Then the patient continued to receive radiation therapy after reoperation but still relapsed after 8 months. The condition may be related to the patient's pathologic subtype of fibrosarcoma DFSP. Another patient with recurrent DFSP had a huge tumor in the abdominal wall that spread throughout the abdominal cavity not long after the operation. Unfortunately, the patient did not undergo adjuvant therapy for a variety of reasons, and the tumor ultimately claimed his life 24 months after surgery.

Finally, the shortcomings of this study need to be analyzed. The study was a small sample size single‐center retrospective study with a single surgical method, which may have information bias. In addition, the follow‐up period was not long enough. This may have made the postoperative recurrence rate less accurate. A multicenter prospective study which includes more cases and a longer duration of follow‐up should be proposed in the future.

In conclusion, early WLE with a negative margin can result in a more favorable prognosis for classic DFSP. The use of flaps to repair the wound can achieve a better appearance and function. For DFSP patients who cannot be radically resected and have multiple recurrences or distant metastases, other antitumor treatments such as radiation therapy or targeted therapy need further exploration. The study may guide clinicians with the appropriate and effective treatment of DFSP patients and provide inspiration for the repair of soft tissue defects after WLE. In addition, the study discusses adjuvant therapy for DFSP, which may provide an alternative for inoperable patients.

## Author Contributions

Bing‐Jie Zhou and Hai‐Yan Zhong performed the research, collected the data, and wrote the paper. Bing‐Jie Zhou, Hai‐Yan Zhong, and Yong Chen designed the research study. Qian Wang, Min Wang, and Ming‐Li Zou polished the paper. Si‐Ming Yuan gave final approval of the version to be published.

## Ethics Statement

Ethical approval was obtained from the Committee of Clinical Investigation in Jinling Hospital.

## Conflicts of Interest

The authors declare no conflicts of interest.

## Data Availability

All data that support the findings of this study are included in this manuscript and its supplementary information files.
